# Circular RNA in cervical cancer: Fundamental mechanism and clinical potential

**DOI:** 10.1016/j.ncrna.2023.11.009

**Published:** 2023-11-18

**Authors:** Sema Begliarzade, Albert Sufianov, Tatiana Ilyasova, Alina Shumadalova, Rinat Sufianov, Ozal Beylerli, Zhongrui Yan

**Affiliations:** a Department of Gynecology, Tianjin Baodi Hospital, Baodi Clinical College of Tianjin Medical University, Tianjin, 301800, China; b Department of Oncology, Radiology and Radiotherapy, Tyumen State Medical University, 54 Odesskaya Street, 625023, Tyumen, Russia; c Educational and Scientific Institute of Neurosurgery, Рeoples’ Friendship University of Russia (RUDN University), Moscow, Russia; d Department of Neurosurgery, Sechenov First Moscow State Medical University (Sechenov University), Moscow, Russia; e Department of Internal Diseases, Bashkir State Medical University, Ufa, Republic of Bashkortostan, 450008, Russia; f Department of General Chemistry, Bashkir State Medical University, Ufa, Republic of Bashkortostan, 3 Lenin Street, 450008, Russia; g Department of Neurooncology, N. N. Blokhin National Medical Research Center of Oncology, Ministry of Health of the Russian Federation, Moscow, Russia; h Central Research Laboratory, Bashkir State Medical University, Ufa, Republic of Bashkortostan, 3 Lenin Street, 450008, Russia

**Keywords:** circRNAs, CC, Biomarker, Therapy, Oncogenesis, Mechanism

## Abstract

CC (CC) remains a significant global health concern, imposing a substantial health burden on women worldwide due to its high incidence and mortality rates. To address this issue, there is a need for ongoing research to uncover the underlying molecular mechanisms of CC and to discover novel diagnostic and therapeutic strategies. Recent progress in non-coding RNAs (ncRNAs) has opened new avenues for investigation, and circular RNAs (circRNAs) have emerged as molecules with diverse roles in various cellular processes. These circRNAs are distinct in structure, forming a closed loop, setting them apart from their linear counterparts. They are intricately involved in regulating different aspects of cellular functions, particularly in cell growth and development. Remarkably, circRNAs can have varying functions, either promoting or inhibiting oncogenic processes, depending on the specific cellular context. Recent studies have identified abnormal circRNAs expression patterns associated with CC, indicating their significant involvement in disease development. The differing circRNAs profiles linked to CC present promising opportunities for early detection, precise prognosis evaluation, and personalized treatment strategies. In this comprehensive review, we embark on a detailed exploration of CC-related circRNAs, elucidating their distinct roles and providing insights into the intricate molecular mechanisms governing CC's onset and progression. A growing body of evidence strongly suggests that circRNAs can serve as valuable biomarkers for early CC detection and hold potential as therapeutic targets for intervention. By delving into the complex interplay between circRNAs and CC, we are paving the way for innovative, individualized approaches to combat this serious disease, with the goal of reducing its impact on women's health globally and improving patient outcomes. As our understanding of circRNAs in the context of CC continues to deepen, the outlook for breakthroughs in diagnosis and treatment becomes increasingly promising.

## 1 Introduction

Cervical cancer (CC) remains a significant global health challenge, imposing a substantial burden on women's health worldwide [1]. Despite substantial progress in prevention and early detection through methods like CC screening and the availability of the human papillomavirus (HPV) vaccine, CC remains a pressing issue. It ranks as the fourth most frequently diagnosed cancer among women and holds the somber distinction of being the fourth leading cause of cancer-related deaths in women. Unfortunately, there are significant geographical disparities in the incidence and mortality rates associated with CC, with developing countries bearing the brunt of this burden. In countries such as Iran and India, CC is alarmingly prevalent. While medical advancements have led to various treatment options for CC, including radical trachelectomy, pelvic lymph node dissection, radiotherapy, chemotherapy, immunotherapy, and targeted therapy, improving survival rates remains an ongoing challenge [2,3]. The complexity of CC, its propensity for recurrence, metastasis, and resistance to conventional therapies underscore the urgent need for innovative approaches in the realms of diagnosis, treatment, and management [4]. Non-coding RNAs (ncRNAs), once regarded as “junk DNA,” have emerged as critical regulators of cellular processes, influencing vital functions like proliferation, differentiation, and gene expression [5]. Within the vast landscape of ncRNAs, we find microRNAs (miRNAs), small nucleolar RNAs (snoRNAs), long non-coding RNAs (lncRNAs), and circular RNAs (circRNAs), with circRNAs taking center stage as intriguing players in the ncRNA arena [[6], [7], [8]]. What sets circRNAs apart is their unique circular structure, formed through a fascinating process known as “backsplicing.” CircRNAs exhibit developmental regulation, tissue-specific expression, and cell-type specificity, making them captivating candidates for in-depth disease research [9]. Recent years have witnessed the revelation of circRNAs' involvement in various diseases, including cancer. Their roles extend beyond acting as miRNA sponges, encompassing influence over RNA-binding proteins (RBPs) transport within cells and modulation of parental gene expression [10]. However, the precise mechanisms by which circRNAs orchestrate their roles in CC and the full spectrum of therapeutic potential they hold are still subjects of active investigation. In this comprehensive review, we embark on a thorough exploration of the burgeoning field of circRNAs in CC [11]. Our journey takes us through their roles in tumorigenesis, intricate interactions with miRNAs, impact on gene expression, modulation of RBP transport, and their tantalizing potential as therapeutic targets. By summarizing emerging evidence and proposing novel insights into how circRNAs shape the landscape of CC, we aim to contribute to a deeper understanding of this multifaceted disease and advance the prospects for enhanced diagnosis, more accurate prognosis, and innovative treatment strategies. Our mission is not just to elucidate the role of circRNAs in CC but also to illuminate a path toward groundbreaking therapeutic interventions that have the potential to alleviate the burden of this devastating disease on women's health across the globe. The promise of circRNAs as biomarkers and therapeutic targets offers a beacon of hope in the fight against CC. Continued exploration of their multifaceted roles holds the key to unlocking breakthroughs in CC management, potentially revolutionizing how we approach this global health challenge and, ultimately, improving the lives of countless women.

## 2 CircRNAs biogenesis

**Fig. 1:**
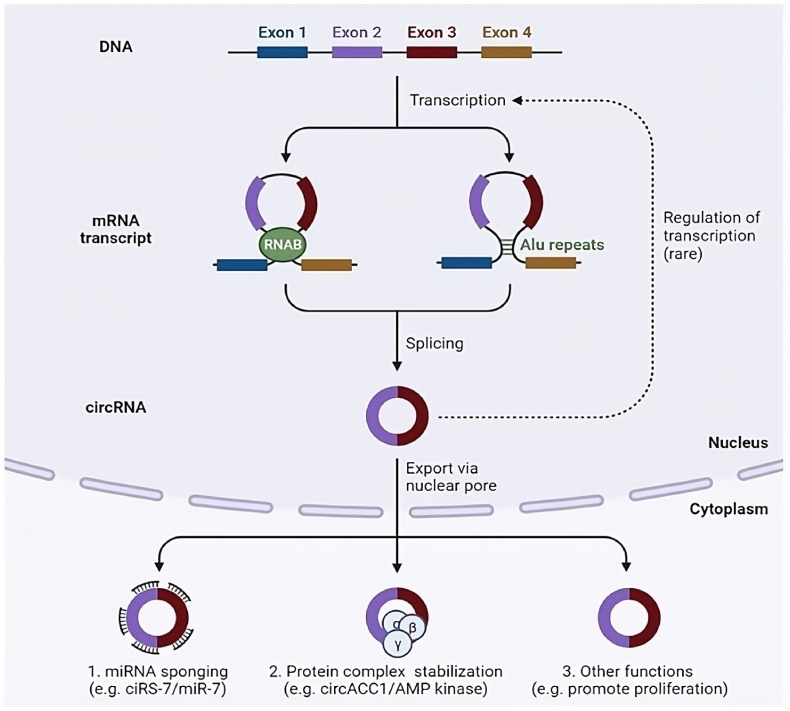
Circular RNA (cicrRNA) biogenesis and function in cancer. CirRNAs are formed from the precursor microRNAs (pre-miRNAs) transcript by backsplicing. Thus, circRNAs are produced as by-products of canonical splice sites and therefore depend on the canonical splicing machinery, which is generally inefficient for generating circRNAs. The loop (ring) can be mediated by base pairing of Alu repeats or other inverted repeat elements located in the source and terminal introns.

**Fig. 2:**
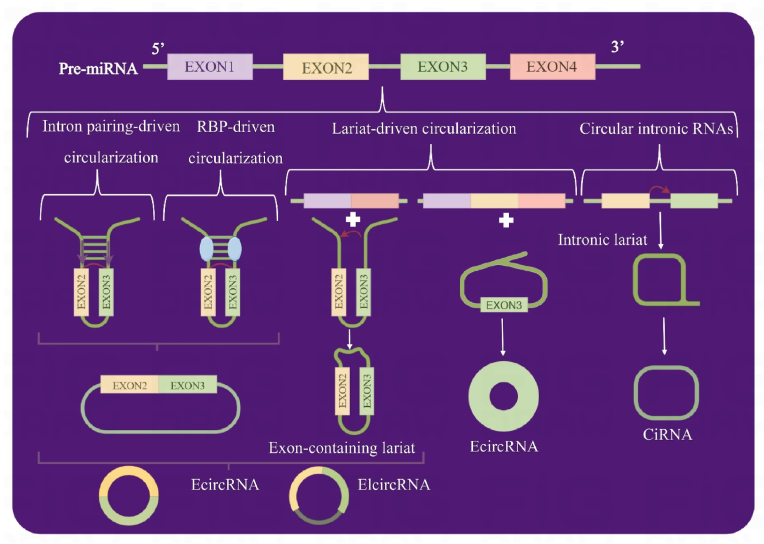
Types of circular RNAs (circular RNAs) and their characteristics. A) Exonic circRNAs (ecircRNAs) arise from back-splicing events involving exonic sequences. B) Intronic circRNAs (ciRNAs), the result of intronic sequences undergoing circularization, ciRNAs add a layer of complexity to the circRNA landscape. C) Exon-intron circRNAs (EliciRNAs) are fascinating subtype that emerges from the circularization of exons while retaining intronic segments.

The fascinating world of circRNAs extends beyond their unique closed circular structure. These enigmatic molecules, defined by their covalently linked 3′ and 5′ ends, exhibit a remarkable resilience that sets them apart from other RNA species (Fig. 1) [12]. Born primarily from pre-mRNA splicing events, circRNAs owe their exceptional stability to their circular configuration, rendering them impervious to degradation by RNases [13,14]. Diverse in their origins, circRNAs can be neatly categorized into three primary types (Fig. 2) [15].

Within the intricate cellular milieu, circRNAs exhibit distinct subcellular distributions. Intronic circRNAs (ciRNAs) and exon-intron circRNAs (EliciRNAs), with their distinctive nuclear localization, contrast exonic circRNAs (EcircRNAs), which predominantly inhabit the cytoplasm [16]. To elucidate the formation of circRNA loops, three predominant models emerge: lariat-driven, intron-pairing-driven, and RBP-driven circularization. These models encompass two fundamental mechanisms: direct back-splicing and exon skipping [12]. In the lariat-driven circularization model, where exon skipping takes center stage, a splice site situated 30 nucleotides upstream of an exon fuse with a site 50 nucleotides downstream. This intricate process culminates in exon-skipping and the creation of an RNA lariat that encapsulates multiple exons and introns. Subsequent removal of introns yields EcircRNAs [13]. In addition, exons can enter circularization while retaining introns, giving rise to EliciRNAs [17]. On the other hand, the intron-pairing- and RBP-driven circularization models, underpinned by direct back-splicing, hinge on reverse complementary sequences, including Alu repeats, strategically positioned in the upstream and downstream introns. These sequences facilitate the proximity of splice donor and acceptor sites, culminating in the formation of a loop. *Trans*-acting activator RBP further enhance this process by binding to the flanking introns [[18], [19], [20]]. Delving into the intricate realm of circular intron RNA biogenesis, we encounter conserved motifs at both ends, notably a 7-nt GU-rich element near the 5′ splice site and an 11-nt C-rich element near the branch-point site. These elements unite, preventing introns from forming branched structures and instead encouraging the emergence of a stable circular structure. The 3′ end of the intron is then strategically relocated to the branch point, giving rise to a resilient circular configuration [21]. CircRNAs, characterized by their widespread presence across diverse human cell types, bring with them the intrigue of cell type-specific expression patterns. Cells with a limited capacity for proliferation, such as cardiomyocytes, often exhibit higher circRNAs expression levels. This contrasts with more rapidly proliferating cell types, such as those found in the liver [18]. The notable abundance of circRNAs in specific tissues largely stems from their accumulation, a direct consequence of their exceptional stability. These molecules, marked by the absence of free ends, boast enhanced stability and a remarkable resistance to degradation by RNase R when compared to their linear RNA counterparts [22]. The allure of circRNAs extends beyond their intrinsic qualities. They hold the promise of serving as novel biomarkers across a spectrum of medical conditions. From essential hypertension [23] to inflammatory bowel disease [24] and a diverse array of cancer types [25], circRNAs are emerging as potent candidates for diagnosis, prognosis, and even therapeutic intervention. In hepatocellular carcinoma, for instance, hsa_circ_0001649 has outshone the established biomarker alpha fetoprotein in terms of sensitivity and specificity [26]. Similarly, hsa_circ_025016 stands as a potential plasma biomarker for predicting postoperative atrial fibrillation [27]. As we delve deeper into the burgeoning field of circRNAs, their multifaceted roles and potential applications continue to unfold, promising a wealth of opportunities for advancements in biomedical research and clinical practice.

## 3 CircRNAs functions

The intricate world of circRNAs is continually unfolding, revealing a plethora of functions that have far-reaching implications in the realm of molecular biology. These versatile molecules, with their multifaceted roles, present an intriguing tapestry of regulatory mechanisms, opening exciting avenues for further exploration and research. One of the most prominent roles that circRNAs play is that of miRNA sponging, a mechanism that has gained substantial attention, particularly in the context of cancer. CDR1as/ciRS-7, a well-studied circRNA, has established itself as a formidable miRNA sponge, effectively curbing tumor growth across various cancer types, including breast cancer (BC), colorectal cancer, gastric cancer (GC), and CC [[28], [29], [30], [31]]. Similarly, circHIPK3, another circRNA that has undergone extensive scrutiny, emerges as a significant player in anti-oncogenic processes. By sequestering miR-558, circHIPK3 exerts a brake on malignant characteristics in bladder cancer cells, resulting in diminished migration, invasiveness, and angiogenesis [32]. Furthermore, circHIPK3's capacity to impede proliferation, migration, and invasiveness in osteosarcoma cells settings underscores its multifunctional nature [33]. Additionally, circHIPK3 has been implicated in promoting proliferation and progression in gallbladder and lung cancer cells, potentially through its interaction with miR-124 [34,35]. These intriguing findings illuminate the Janus-faced character of circRNAs, revealing their ability to act as both guardians and instigators in cellular processes. Another captivating facet of circRNA function revolves around their interactions with RBP. The outcomes of these interactions are highly context-dependent, relying on the specific partnership between proteins and circRNAs, ultimately leading to diverse regulatory effects. For instance, circFoxo3 forms a complex with p21 and cyclin dependent kinase 2 (CDK2), known as the circFoxo3/CDK2/p21 axis, which effectively halts the progression from the G1 to the S phase of the cell cycle [36]. Beyond this, circFoxo3 has been shown to bind p53 and mouse double minute 2 homolog, instigating ubiquitination and subsequent degradation facilitated by p53 [37]. Notably, circMbl interacts with mannose-binding lectin (MBL), and emerging evidence suggests that MBL levels can impact circMbl biosynthesis through a feedback loop mechanism. Moreover, elevated MBL protein levels may influence MBL pre-mRNA translation into circMbl, while circMbl, in turn, appears to affect the availability of MBL [38]. Several other circRNAs have been identified for their capacity to interact with multiple proteins, thus playing multifaceted roles in diverse cellular processes [39]. While a majority of circRNAs involved in gene expression regulation through miRNA sponging predominantly reside in the cytoplasm, recent research has unveiled a subset of nuclear circRNAs that exert control at the transcriptional level. circEIF3J and circPAIP2, found within the nucleus, facilitate the expression of parental genes by engaging with RNA polymerase II (Pol II), U1 small nuclear RNA, and several promoter regions [40]. Additionally, ciRNAs, as identified by Zhang et al., have been recognized as positive regulators of RNA polymerase-II transcription, actively promoting the expression of parental genes [21]. Other nuclear circRNAs, including ciRNA from the gene ankyrin repeat domain 52 (ANKRD52) (ci-ankrd52) and ciRNA from the gene Sirtuin 7(SIRT7) (ci-SIRT7), interact with Pol II, thereby modulating the transcriptional rate of parental genes by accumulating at active transcription sites [21]. Furthermore, circRNA FECR1 binds to the promoter region and recruits translocation methylcytosine dioxygenases (TET1) DNA demethylase, thereby regulating friend leukemia integration 1 transcription factor (FLI1) gene expression and inducing DNA demethylation [41]. These diverse circRNAs encompass various origins, spanning intronic, exonic, and exon-intron configurations. Intriguingly, despite their categorization as ncRNAs, a growing body of evidence underscores the involvement of circRNAs in protein translation. Unlike their linear RNA counterparts, circRNAs typically lack 7-methylguanosine cap structures and poly(A) tails, features that usually render them impervious to ribosomal recognition and subsequent translation into proteins. However, the discovery of internal ribosome entry sites has unveiled the translational potential of circRNAs [42]. Notably, circ-ZNF609, known for its role in regulating myoblast proliferation, features an open reading frame amenable to cap-independent protein translation [43]. Similarly, circ-FBXW7, abundantly expressed in the normal human brain, encodes a novel protein that governs cancer cell proliferation and cycling [44]. Moreover, circ-SHPRH encodes SHPRH-146aa, a key regulator of cancer cell proliferation and tumorigenicity in vitro [45]. It is worth noting that only a limited number of circRNAs have been confirmed to undergo translation into proteins, warranting further exploration in this intriguing domain. As our understanding of circRNAs deepens, their multifaceted functions continue to unveil new dimensions in the intricate landscape of cellular biology. These remarkable molecules hold tremendous potential, with implications ranging from fundamental research to the development of clinical applications, particularly in the context of cancer and beyond. The intricate interplay of circRNAs in cellular processes presents an exciting and evolving field of study, ripe with opportunities for discovery and innovation.

## 4 CircRNAs and CC

CC, a pressing global health concern, ranks as the fourth most prevalent cancer among women and poses a significant threat to lives (Table 1) [46].

**Table 1:** Functional characterization and expression of circular RNAs (circRNAs) in cervical cancer (CC).

circRNAs	Expression	Type of role	Binding miRNA	Targets	Biological effect	References
circ_0018289	Up	Oncogenic	miR-497	–	Promotes CC cells proliferation migration and invasion	[47]
circ_0067934	Up	Oncogenic	miR-545	EIF3C	Promotes CC progression and metastasis	[48]
circ_0023404	Up	Oncogenic	miR-136	TFCP2 and YAP1	Promotes tumorigenesis.	[49]
					Poor prognosis	
circRNA-000284	Up	Oncogenic	miR-545	Snail-2	Promotes CC cell proliferation and invasion	[50]
circRNA8924	Up	Oncogenic	miR-518d-5p/519–5p	CBX8	Promotes CC cells proliferation, migration and invasion. Associated with tumor size, FIGO staging and myometrial invasion	[51]
circ-ATP8A2	Up	Oncogenic	miR-433	EGFR	Promotes CC progression, lymph node invasion and correlates with FIGO stage	[52]
circ-0000745	Up	Oncogenic	–	E-cadherin	Acts as a tumor promoter and promotes vascular/lymphatic invasion	[53]
circ_0005576	Up	Oncogenic	miR-153–3p	KIF20A	Promotes CC progression, lymph node invasion and correlates with FIGO stage	[54]
circRNA_101308	Down	Tumor suppressive	miR-26a-5p,	–	Inhibits CC progression and deep myometrial invasion and lymph node metastasis	[55]
			miR-196a-5p,			
			miR-196b-5p, miR-335–3p, and miR-1307–3p			
circSLC26A4	Up	Oncogenic	miR-1287–5p	HOXA7	Facilitates cancer progression.	[56]
					Poor prognosis	
circ_0018289	Up	Oncogenic	miR-497	–	Promotes tumorigenesis. Poor prognosis	[57]
circ-ITCH	Down	Tumor suppressive	miR-93–5p	FOXK2	Suppresses cancer cells proliferation and metastasis	[58]

**Abbreviations:** EIF3C, Eukaryote translation initiation factor (eIF) 3 complex; TFCP2, Homo sapiens transcription factor CP2; YAP, Yes-associated protein 1; CBX8, Chromobox 8; FIGO, International Federation of Gynecology and Obstetrics; EGFR, Epidermal growth factor receptor; KIF20A, Kinesin family member 20A; HOXA7, Homeobox A7; FOXK2, Forkhead box K2; -, not reported.

**Fig. 3:**
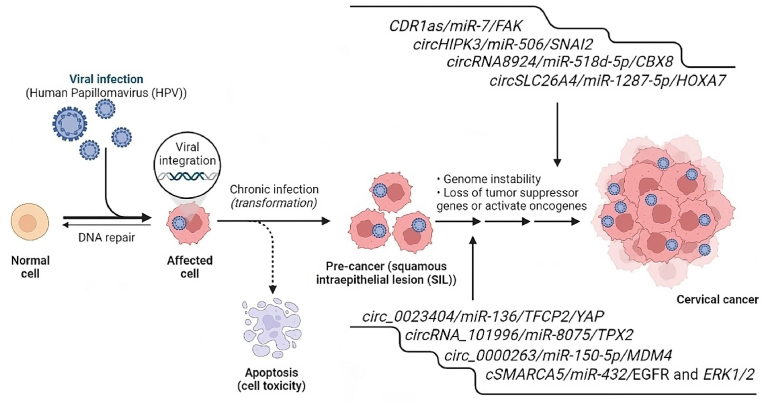
Viral (Human Papillomavirus (HPV)) oncogenesis in CC (CC) and role circRNAs in the development of HPV-associated CC. During the malignant transformation of the cervical epithelium, the persistence of HPV leads to characteristic changes in the expression profile of circRNAs with oncogenic properties and, accordingly, their regulatory activity. Changes in the cellular circRNAs profile during HPV infection are mainly mediated by the activity of the E6 and E7 genes.

Multiple well-established risk factors, including smoking, HPV infection, organ transplantation, a family history of CC, and continuous oral contraceptive use, contribute to an individual's susceptibility to CC [59]. However, from both epidemiological and molecular perspectives, it is persistent HPV infection that emerges as the primary instigator of CC. Among the over 100 known HPV types, those classified as high-risk due to their oncogenic potential stand out [60]. Notably, HPV type 16 is responsible for approximately 55–60 % of CC cases, with HPV type 18 ranking as the second most carcinogenic type in this context (Fig. 3) [61].

**Fig. 4:**
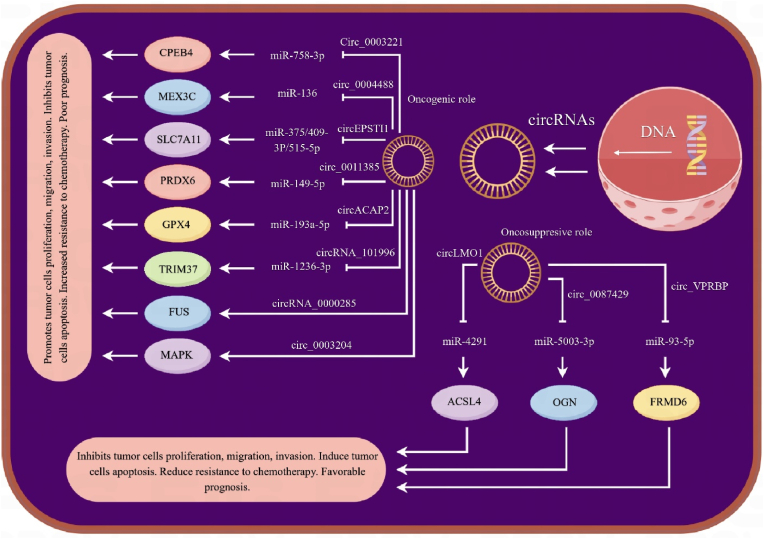
The tumorigenic of circular RNAs (circRNAs)-micoRNAs (miRNAs)-messenger RNAs (mRNAs) axis in cervical cancer (CC) cell. Some circRNAs acting as miRNA sponges have oncogenic and tumor-suppressive properties.

In the intricate web of CC development, several molecules are overexpressed, including phosphoinositide 3-kinases (PI3Ks), epidermal growth factor receptor (EGFR), β-catenin, extracellular-signal-regulated kinase 1/2 (ERK1/2), and antiapoptotic factors such as B-cell lymphoma 2 (Bcl-2). Each of these elements represents a potential target for therapeutic interventions [62]. Currently, a multitude of studies have delved into the roles of circRNAs and their potential mechanisms in CC. These investigations have unveiled that circRNAs assume diverse roles in the progression of CC, with miRNA sponging emerging as a particularly pivotal mechanism. In this comprehensive overview, we provide an in-depth exploration of some of these studies conducted to date, shedding light on the intricate mechanisms through which circRNAs contribute to CC development. In a recent study of CC tissues, researchers employed microarray analysis to scrutinize the expression of circRNAs. Their findings unveiled 45 differentially expressed circRNAs, with has_circ-0018289 emerging as the most prominently upregulated. This study presented compelling evidence that the depletion of has_circ-0018289 leads to the inhibition of tumor cell proliferation, invasion, and migration. Furthermore, it unveiled that has_circ-0018289 functions as a miRNA sponge, directly interacting with miR-497 [63]. Tian and Liang et al., embarked on an exploration of the mechanism of action of another circRNA, circSMARCA5 (also known as has_circ_0001445), in the context of CC progression [64]. Their meticulous investigation revealed that circSMARCA5 expression is downregulated in CC cells, and its augmentation brings about the suppression of tumor cell proliferation, invasion, migration, and the induction of cell cycle arrest. Furthermore, it was elucidated that circSMARCA5 engages in an intricate dance with miR-620, leading to the downregulation of miR-620 expression. This groundbreaking study postulates that circSMARCA5 could serve as a promising therapeutic focal point in CC by orchestrating the regulation of miR-620 expression. Yet another circRNA that takes center stage in CC progression is has_circ_0023404. It has come to light that this circRNA is conspicuously overexpressed in CC cells in comparison to normal cells. Its upregulation was closely linked to a grim prognosis in CC patients. Intriguingly, the silencing of has_circ_0023404 exerted a profound impact by markedly suppressing proliferation, impeding cell cycle progression, thwarting migration, and curtailing metastasis of CC cells. The key to its influence lies in its role as a miR-136 sponge, leading to the heightened expression of transcription factor CP2 (TFCP2) and the consequent activation of the YAP signaling pathway—an orchestration that ultimately culminates in the promotion of CC development [65]. A relatively recent inquiry delved into the expression patterns and mechanisms of action of has_circ_0000263 in CC. The meticulous examination illuminated the fact that has_circ_0000263 exhibits an upregulation in CC and serves as a miR-150–5p sponge. In an intriguing twist, it was unveiled that has_circ_0000263 exerts an impact on the expression of the p53 gene. Pertinently, the knockdown of has_circ-0000263 resulted in a dramatic inhibition of cell growth and migration [66]. Enter circRNA-000284, yet another participant in the intricate dance of CC progression. This circRNA emerged as a potent suppressor of cell proliferation, invasion, and an inducer of cell cycle arrest in the G0/G1 phase. Its modus operandi hinges on its capacity to act as a miR-506 sponge, a pivotal role in the intricate machinery by which it directly targets Snail-2, thereby stymying cancer progression. The promise of silencing circRNA-000284 as a therapeutic strategy in CC treatment shines brightly [67]. Liu et al. embarked on a quest to illuminate the role of circRNA8924 in the context of CC [68]. Their exhaustive examination bore fruit by revealing the overexpression of circRNA8924 in CC compared to normal tissues. Significantly, a correlation was unearthed between the levels of circRNA8924 expression and tumor size and invasion. This circRNA8924, once characterized, was found to wield considerable influence, promoting proliferation, migration, and invasion of cervical tumor cells. This discovery opens new vistas wherein circRNA8924 could potentially serve as both a diagnostic biomarker and a tantalizing therapeutic target in the realm of CC. Bioinformatic analysis, a powerful tool in the modern scientific arsenal, pointed the way to has_circRNA_101996, which emerges as a pivotal player in CC. This circRNA stands as an entity overexpressed in CC cells and bears a positive association with crucial clinical parameters such as cancer stage, tumor size, and lymph node metastasis. Notably, has_circRNA_101996 earned its place in the spotlight as a miR-8075 sponge, which, in turn, targets TPX2 in CC. The intricate web thus woven reveals that has_circRNA_101996 masterminds the downregulation of miR-8075 and TPX2, resulting in an orchestration that ultimately promotes CC development [69]. Circ-ATP8A2, another circRNA, steps into the limelight, exhibiting an upregulation in CC cells. Recent investigations have unveiled the role of circ-ATP8A2 as a sponge for miR-433. In this capacity, it exerts its influence by inhibiting posttranscriptional EGFR expression, thereby setting the stage for the relentless progression of CC [70]. And then, there's circ_0067934, a circRNA that has only recently emerged from the shadows. This circRNA has made its presence felt by aligning itself with advanced CC stages, lymph node metastasis, and dismal prognoses for CC patients. Its expression levels shine a spotlight on these aggressive features, forging a correlation that demands attention. Significantly, the suppression of circ_0067934 has proven to be a potent tool, leading to the inhibition of proliferation, invasion, and the epithelial-mesenchymal transition (EMT) in cancer cells. This newly discovered circRNA, identified as a miR-545 sponge, emerges as a gatekeeper inhibiting CC development. As such, the silencing of circ_0067934 emerges as a tantalizing prospect that could potentially deliver therapeutic benefits in the treatment of CC [71]. In the report by Mao et al., circEIF4G2 steps onto the stage, significantly upregulated in CC cells [72]. Its absence results in the suppression of various malignant properties displayed by CC cells. Unveiling yet another layer of complexity, circEIF4G2 reveals itself as a miR-218 sponge, with miR-218 poised to target homeobox A1 (HOXA1) expression. This orchestration positions circEIF4G2 as a mastermind driving CC progression through the miR-218/HOXA1 axis. In summary, these findings underscore the pivotal roles played by circRNAs in the intricate landscape of CC development (Fig. 4). These circRNAs, with their multifaceted mechanisms, not only offer insight into the disease's molecular intricacies but also serve as promising candidates for diagnosis and potential therapeutic intervention in the quest to combat CC effectively. This burgeoning field of research holds the promise of unveiling additional layers of complexity in the molecular mechanisms underpinning CC, offering hope for improved outcomes and enhanced quality of life for individuals grappling with this formidable disease.

## 5 Perspectives and limitations

The potential synergy between miRNAs and circRNAs represents a promising frontier in the field of epigenetics. It is crucial to emphasize the need for additional experiments, not only in the context of CC but across various cancer types. These experiments are imperative to unravel the distinct and collaborative roles played by these two ncRNA entities. The outcomes of such investigations are poised to make substantial contributions to the forthcoming comprehensive research article. Currently, there is a growing enthusiasm for advancing nanocarrier technologies designed to co-deliver both small molecules and genes for combating cancer. Gene-based therapies, involving small interfering RNA (siRNA) and miRNA, have emerged as innovative strategies poised to enhance existing cancer treatment modalities [73]. Recent studies have provided compelling evidence for the efficacy of co-delivering siRNA targeting the interleukin 17 receptor B (IL17RB) gene alongside the chemotherapeutic agent doxorubicin (DOX) using nanoparticles in the context of BC treatment. This approach demonstrates a remarkable reduction in the relative gene expression levels of nuclear factor kappa-light-chain-enhancer of activated B cells (NF-κB) and Bcl-2, accompanied by the induction of apoptosis and the inhibition of migration in BC cells [74]. Moreover, within the realm of CC, nanoparticles loaded with gold-ursolic acid have been investigated as potential therapeutics, capitalizing on ursolic acid's capacity to modulate cellular processes and the heightened nanoparticle uptake [75]. These explorations strongly suggest that nanoparticle-mediated co-delivery systems hold immense promise in the treatment of CC, extending to a wide spectrum of therapeutic molecules, including siRNAs, miRNAs, or circRNAs [76]. This avenue of research merits substantial attention. The recent emergence of genome-editing technologies has ushered in a new era wherein the human genome sequence can be precisely manipulated for therapeutic purposes. This transformative capability encompasses the correction of disease-causing mutations, the targeted addition of therapeutic genes at specific genomic loci, and the selective removal of deleterious genes or genomic sequences. Notably, CRISPR/Cas9 technology has demonstrated remarkable potential in rectifying or deactivating mutations in animal models afflicted by monogenic human diseases [77]. Within the domain of CC therapeutics, gene therapy has been both explored and applied. Specifically, genes such as tumor necrosis factor-related apoptosis-inducing ligand (TRAIL) and/or endostatin have been effectively delivered using nanoparticles for CC treatment [78]. This approach exhibits substantial potential as an ideal candidate for in vivo cancer gene delivery therapy. Given the central role that circRNAs play in CC tumorigenesis, it is conceivable to hypothesize that gene therapy targeting specific gene loci associated with circRNAs could emerge as an innovative therapeutic approach for CC. However, it is vital to acknowledge that this hypothesis is grounded in existing research and underscores the need for further investigations to delve into this nascent field. Recent research endeavors have cast a spotlight on exosomes, small extracellular vesicles spanning 30–100 nm in size. Exosomes are characterized by a lipid bilayer and well-defined spherical morphology, and they play a pivotal role in the transport of intercellular materials and communication between proximal and distant cells [79]. Intriguingly, studies have unveiled the abundance of exosomes in cervicovaginal lavage specimens obtained from women afflicted with CC [80]. Furthermore, deeper inquiries into exosomes have unveiled their enrichment in lncRNAs originating from CC cells. This enrichment is underscored by the prominent expression of lncRNAs such as HOX antisense intergenic RNA (HOTAIR), metastasis-associated lung adenocarcinoma transcript 1 (MALAT1), and maternally expressed 3 (MEG3) within CC-derived exosomes retrieved from cervicovaginal lavage samples [81]. Additionally, the specific enrichment of miR-221–3p has been noted within exosomes secreted by cervical squamous cell carcinoma cells. In this context, miR-221–3p has been shown to facilitate the migration and tube formation of human lymphatic endothelial cells, thereby promoting lymphangiogenesis and lymph node metastasis [82]. Regarding circRNAs, they have been observed to be particularly abundant within exosomes, often surpassing their cellular counterparts in terms of quantity. This suggests an active cellular packaging mechanism for circRNAs into exosomes, highlighting their role in intercellular communication [83]. Exosome-mediated delivery of circRNAs has been found to be instrumental in facilitating cell-to-cell communication within various cancer contexts [84]. For instance, circRNAs present in plasma exosomes have demonstrated unique expression patterns in GC, actively participating in white adipose tissue browning by activating PR domain-containing 16 (PRDM16) and suppressing miR-133 [85]. Therefore, it is plausible to speculate that extracellular circRNAs transported via exosomes in CC have the potential to modulate specific functions in recipient cells by influencing related miRNAs, thereby orchestrating the regulation of CC progression. However, it is imperative to note that related experiments in this domain are scarce, necessitating further research efforts to deepen our comprehension of this promising field. In summary, the intricate interplay between miRNAs, circRNAs, and various molecular mechanisms holds tremendous promise within the realm of cancer research, particularly in the context of CC. Thus, the imperative lies in conducting additional experiments encompassing CC and various other cancer types to unveil the precise roles of these ncRNAs. Additionally, the utilization of nanoparticles for the co-delivery of therapeutic agents and the potential of gene therapy, particularly targeting gene loci associated with circRNAs, warrant thorough exploration. Furthermore, the role of exosomes in the transport of circRNAs and their influence on intercellular communication within CC necessitate further investigation. These diverse avenues of research have the potential to advance our understanding of cancer biology and pave the way for innovative therapeutic strategies that hold promise for the future.

CC continues to pose a significant and persistent threat to women's health due to its high incidence rates. This underscores the urgent need for intensified research efforts and focused attention on this pressing issue. Interestingly, circRNAs have emerged as intriguing and promising elements in the context of CC. They hold distinctive potential as biomarkers, capable of significantly enhancing the precision of diagnosis and the accuracy of prognostic assessments. For instance, the overexpression of a specific circRNA, hsa_circ_0023404, has been linked to the overactivation of the YAP pathway, a pivotal contributor to the initiation and progression of CC [53]. Nevertheless, this revelation raises thought-provoking questions: do circRNAs undergo complex regulation by multiple miRNAs, thereby exerting a multifaceted influence on downstream gene expression? How does interplay and crosstalk impact circRNA expression during the development of CC? Future investigations are poised to illuminate the intricacies of these processes. It is crucial to underscore that, while deciphering the mechanistic roles of circRNAs is pivotal, an equivalent emphasis must be placed on unraveling their clinical significance in the context of CC. With the continuous advancement of next-generation sequencing and microarray technologies, our comprehension of the enigmatic roles played by circRNAs in CC, as well as various other diseases, is progressively unfolding. In clinical practice, specialists are progressively approaching a deeper understanding of how to strategically manipulate circRNAs for therapeutic interventions against a spectrum of diseases. Consequently, ongoing, and vigilant monitoring of developments in this field is imperative, as it holds the promise of not only advancing our scientific knowledge but also enhancing our ability to manage and treat CC effectively. CircRNAs, once overlooked and underestimated, have now rightfully assumed their position as a prominent and noteworthy family of ncRNAs. The advent of advanced sequencing techniques and sophisticated bioinformatics tools has revealed an extensive repertoire of circRNAs within human cells, spanning various pathological conditions, with cancer being of particular interest. CC, as a prevalent malignancy among women, has not escaped the scrutiny of circRNA research. These investigative efforts have unveiled substantial differences in the expression profiles of circRNAs between CC cells and their normal counterparts, strongly suggesting that these circRNAs play pivotal roles with biological relevance in this neoplastic disease. One of the predominant mechanisms through which circRNAs exert their influence in CC is through the process of miRNA sponging. Multiple miRNAs have been implicated in the progression of CC, and circRNAs intricately modulate this progression by interacting with and regulating the activity of these miRNAs. It is paramount to acknowledge that while circRNAs exhibit immense promise in the diagnosis and treatment of CC, the precise mechanisms governing their actions remain incompletely understood.

## 6 Conclusion

CircRNAs represent an exciting and promising frontier in the diagnosis and therapeutic intervention of CC, offering new avenues and possibilities for significantly improving patient outcomes. Their multifaceted roles in CC, both mechanistically and clinically, underscore the importance of continued research and exploration in this dynamic field.

## Funding

This work was supported by the Bashkir State Medical University Strategic Academic Leadership Program (PRIORITY-2030).

## CRediT authorship contribution statement

**Sema Begliarzade:** Conceptualization, Writing – original draft. **Albert Sufianov:** Conceptualization, Data curation, Writing – review & editing. **Tatiana Ilyasova:** Formal analysis, Resources. **Alina Shumadalova:** Investigation, Software, Writing – review & editing. **Rinat Sufianov:** Formal analysis, Software, Validation. **Ozal Beylerli:** Supervision. **Zhongrui Yan:** Supervision.

## Declaration of competing interest

Ozal Beylerli is an editorial board member for Non-coding RNA Research and was not involved in the editorial review or the decision to publish this article. All authors declare that there are no competing interests.
